# Supplementary material for: The mRubyFT Protein, Genetically Encoded Blue-to-Red Fluorescent Timer
Source: Int J Mol Sci. 2022 Mar 16;23(6):3208. doi: 10.3390/ijms23063208 (PMC8952768; doi:10.3390/ijms23063208)
Supplement: Supplementary file 1 [file ijms-23-03208-s001.zip › ijms-1646700-supplementary.pdf]

Supplementary Information

The mRubyFT Protein, Genetically Encoded Blue-to-Red Fluorescent Timer

Oksana M. Subach, Aleksandr Tashkeev, Anna V. Vlaskina, Yuliya K. Agapova, Filipp A. Gaivoronskii, Alena Y. Nikolaeva, Olga I. Ivashkina, Konstantin V. Anokhin, Vladimir O. Popov, Konstantin M. Boyko, and Fedor V. Subach

Supplementary Tables and Figures

**Table S1**	**2**
**Table S2**	**3**
**Figure S1**	**5**
**Figure S2**	**6**
**Figure S3**	**7**
**Figure S4**	**8**
**Figure S5**	**9**
**Figure S6**	**10**
**Figure S7**	**11**

Table S1. Characteristics of RubyFT#11-9 timer based on the mRuby2 red fluorescent protein. [a]- extinction coefficients for red-forms were determined by alkaline denaturation method; extinction coefficients for blue-forms were determined by acid denaturation method. [b] - quantum yields (QYs) for blue and red forms were determined relative to mTagBFP2 (QY of 0.64) and mCherry (QY of 0.22), respectively. [c] – brightness was calculated as a product of quantum yield and extinction coefficient relative to the brightness of EGFP protein (QY of 0.6 and extinction coefficient of 56000 M^{-1} cm^{-1}). [d] – characteristic times for the blue- and red-forms of Fast-FT, Medium-FT and mRubyFT#11-9 correspond to the maximum of the blue fluorescence and half of the red fluorescence, respectively, at 37°C. [e] – data from reference [1].

Timer	Form	Abs/Em maxima (nm)	ε ($mM^{-1}cm^{-1}$) [a]	QY [b]	Brightness vs EGFP [c](%)	Characteristic times (h) [d]
FastFT [e]	blue	403/466	49.7	0.3	44	0.25
	red	583/606	75.0	0.09	20	7.1
MediumFT [e]	blue	401/464	44.8	0.41	55	1.2
	red	579/600	73.1	0.08	17	3.9
RubyFT#11-9	blue	402/454	66	0.14	28	2.4
	red	560/592	92	0.308	84	4.9

Table S2. List of primers.

Primer	Primer sequence (5′-3′)
Fw-BglII-(PA)TagRFP	GC TCG AGA TCT ATGGTGTCTAAGGGCGAAGAG
Rv-GFP-EcoRI	GTCGAATTCttacttgtacagctcgtccatgcc
mRubyFT-65	CATTCTTGCCACGTCGTTCMTGTATGGCAGCCGTACTTTTATC
mRubyFT-65-r	GATAAAAGTACGGCTGCCATACAKGAACGACGTGGCAAGAATG
mRubyFT-148	CCAAGGGTTGGGAGCCTWMTACAGAGATGATGTATC
mRubyFT-148-r	GATACATCATCTCTGTAKWAGGCTCCCAACCCTTGG
mRubyFT-165	GTGGTCTGAGGGGATACNNSCATNNSGCACTGAAAGTTGATGG
mRubyFT-165-r	CCATCAACTTTCAGTGCSNNATGSNNGTATCCCCTCAGACCAC
mRubyFT-220-r	GTACAGCTCGTCCATGCCACCAAGCCCGGCGAACTTGGCAACASHGT GTTCGCGTWGTACTACGAACATTTCATTG
mRuby-M65L;	ATTCTTGCCACGTCGTTCcTGTATGGCAGCCGTACTT
mRuby-M65L-r;	AAGTACGGCTGCCATACAgGAACGACGTGGCAAGAAT
mRuby-Q220L;	CAATGAAATGTTCGTAGTACtACGCGAACACGCAGTTGCC
mRuby-Q220L-r	GGCAACTGCGTGTTCGCGTaGTACTACGAACATTTCATTG
mRubyFT-148F	CAAGGGTTGGGAGCCTttTACAGAGATGATGTATC
mRubyFT-148F-r	GATACATCATCTCTGTAaaAGGCTCCCAACCCTTG
mRubyFT-148I	CAAGGGTTGGGAGCCTatTACAGAGATGATGTATC
mRubyFT-148I-r	GATACATCATCTCTGTAatAGGCTCCCAACCCTTG
mRubyFT-167Q	GAGGGGATACACTCATcaGGCACTGAAAGTTGATG
mRubyFT-167Q-r	CATCAACTTTCAGTGCCtgATGAGTGTATCCCCTC
mRubyFT-62S	CTTTGACATTCTTGCCAgcTCGTTCCTGTATGGCAG
mRubyFT-62S-r	CTGCCATACAGGAACGAgcTGGCAAGAATGTCAAAG
mRubyFT-224A	TAGTACTACGCGAACACgcTGTTGCCAAGTTCGCCG

mRubyFT-224A-r	CGGCGAACTTGGCAACAgcGTGTTCGCGTAGTACTA
mRubyFT-224C	TAGTACTACGCGAACACTgTGTTGCCAAGTTCGCCG
mRubyFT-224C-r	CGGCGAACTTGGCAACAcAGTGTTCGCGTAGTACTA
mRubyFT-165N	GTGGTCTGAGGGGATACAaTCATATGGCACTGAAAGTTGATG
mRubyFT-165N-r	CATCAACTTTCAGTGCCATATGAtTGTATCCCCTCAGACCAC
mRubyFT-203Y	GTATCCATGCCGTTGATtACCGCCTGGAAAGGTTAG
mRubyFT-203Y-r	CTAACCTTTCCAGGCGGTaATCAACGGCATGGATAC
mRubyFT-69K	GTTCCTGTATGGCAGCaagACTTTTATCAAGTACC
mRubyFT-69K-r	GGTACTTGATAAAAGTcttGCTGCCATACAGGAAC
RubyFT-NheI2	tccgctagcggtaccggtcgccaccATGGTGTCTAAGGGCGAAGAG
RubyFT-AgeI-r	tgaaccggtcgCTTGTACAGCTCGTCCATG
RubyFT-BglII-r	tcgagatctcctagggagtccggaCTTATACAGCTCGTCCATGCCAC
RubyFT-EcoRI	CAGGAATTCgccaccATGGTGTCTAAGGGCGAAGAG
LSSmSc-XbaI-r	tgatctagattaCTTGTACAGCTCGTCCATG
mCherry-KpnI	TCT GGT ACC ATG GTG AGC AAG GGC GAG

mRubyFT-stop gene:

ATGGTGTCTAAGGGCGAAGAGCTGATCAAGGAAAATATGCGTATGAAGGTGGTCATGGAAGGTTCGGTCAACGGCCACCAATTC
AAATGCACAGGTGAAGGAGAAGGCAATCCGTACATGGGAACTCAAACCATGAGGATCAAAGTCATCGAGGGAGGACCCCTGCCA
TTTGCCTTTGACATTCTTGCCACGTCGTTCCTGTATGGCAGCCGTACTTTTATCAAGTACCCGAAAGGCATTCCTGATTTCTTTAAA
CAGTCCTTTCCTGAGGGTTTTACTTGGGAAAGAGTTACGAGATACGAAGATGGTGGAGTCGTCACCGTCATGCAGGACACCAGCC
TTGAGGATGGCTGTCTCGTTTACCACGTCCAAGTAAGAGGGGTAGACTTTCCCTCCAATGGTCCCGTGATGCAGAAGAAGACCAA
GGGTTGGGAGCCTTCTACAGAGATGATGTATCCAGCAGATGGTGGTCTGAGGGGATACACTCATATGGCACTGAAAGTTGATGGT
GGTGGCCATCTGTCTTGCTCTTTCGTAACAACTTACAGGTCAAAAAAGACCGTCGGGAACATCAAGATGCCCGGTATCCATGCCG
TTGATCACCGCCTGGAAAGGTTAGAGGAAAGTGACAATGAAATGTTCGTAGTACTACGCGAACACTCTGTTGCCAAGTTCGCCGG
GCGTGGTGGCATGGACGAGCTGTACAAGTAA

RubyFT#11-9-stop gene:

ATGGTGTCTAAGGGCGAAGAGCTGATCAAGGAAAATATGCGAATGAAGGTGGTCATGGAAGGTTCGGTCAACGGCCACCA
ATTCAAATGCACAGGTGAAGGAGAAGGCAATCCGTACATGGGAACTCAAACCATGTGGATCAAAGTCATCGAGGGAGGA
CCCCTGCCATTTGCCTTTGACATTCTTGCCACGTCGTTCATGTATGGCAGCCGTACTTTTATCAAGTACCCGAAAGGCATTCC
TGATTTCTTTAAACAGTCCTTTCCTGAGGGTTTTACTTGGGAAAGAGTTACGAGATACGAAGATGGTGGAGTCGTCACCGTC
ATGCAGGACACCAGCCTTGAGGATGGCTGTCTCGTTTACCACGTCCAAGTCAGAGGGGTAAACTTTCCCGCCAATGGTCCC
GTGATGCAGAAGAAGACCAAGGGTTGGGAGCCTAATACAGAGATGATGTTTCCAGCAGATGGTGGTCTGTGGGGATGCAC
TCATAGGGCACTGAAAGTTGATGGTGGAGGCCATCTGTCTTGCTCTTTCGTAACAACTTACAGGTCAAAAAAGACCGTCGG
GAACATCAAGATGCCCGGTATCCATGCCGTTGATCACCGCCTGGAAAGGTTAGAGGAAAGTGACAATGAAATGTTCGTAG
TACAACGCGAATACGCAGTTGCCAAGTTCGCCGGGCTTGGTGGTGGCATGGACGAGCTGTACAAGTAATAA

Figure S1. Nucleotide sequences of the mRubyFT and RubyFT#11-9 proteins.

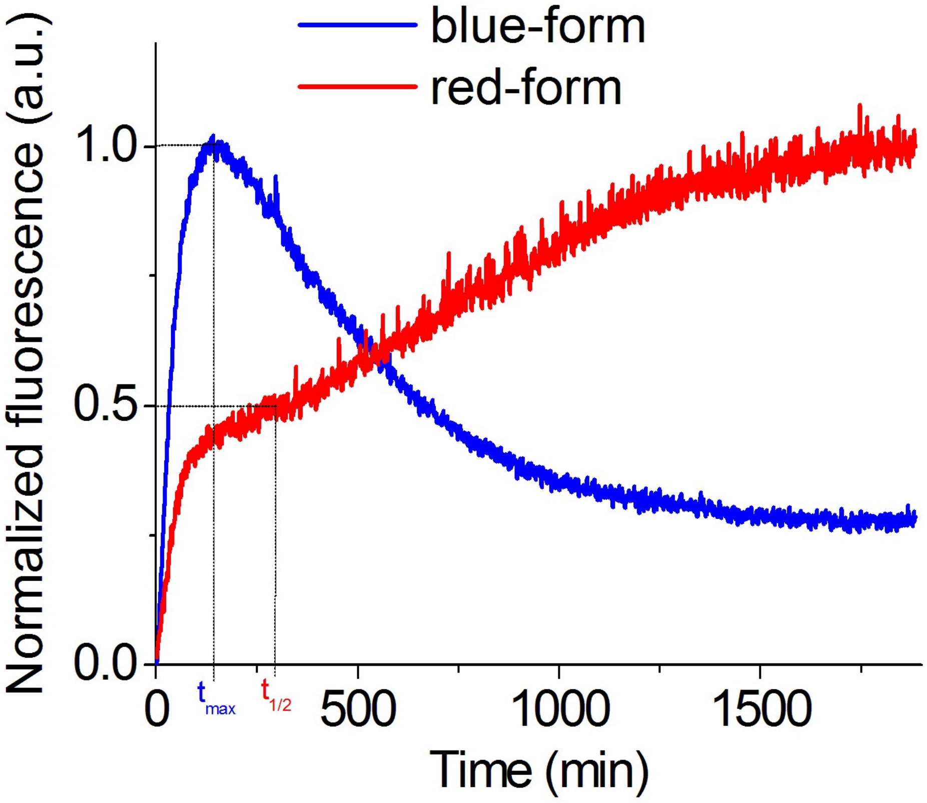

Figure S2. Maturation kinetics of blue and red forms for the purified RubyFT#11-9 timer at 37 °C. The characteristic times are indicated for the blue (t_{max} = 2.4 h) and red ($t_{1/2}$ = 4.9 h) forms.

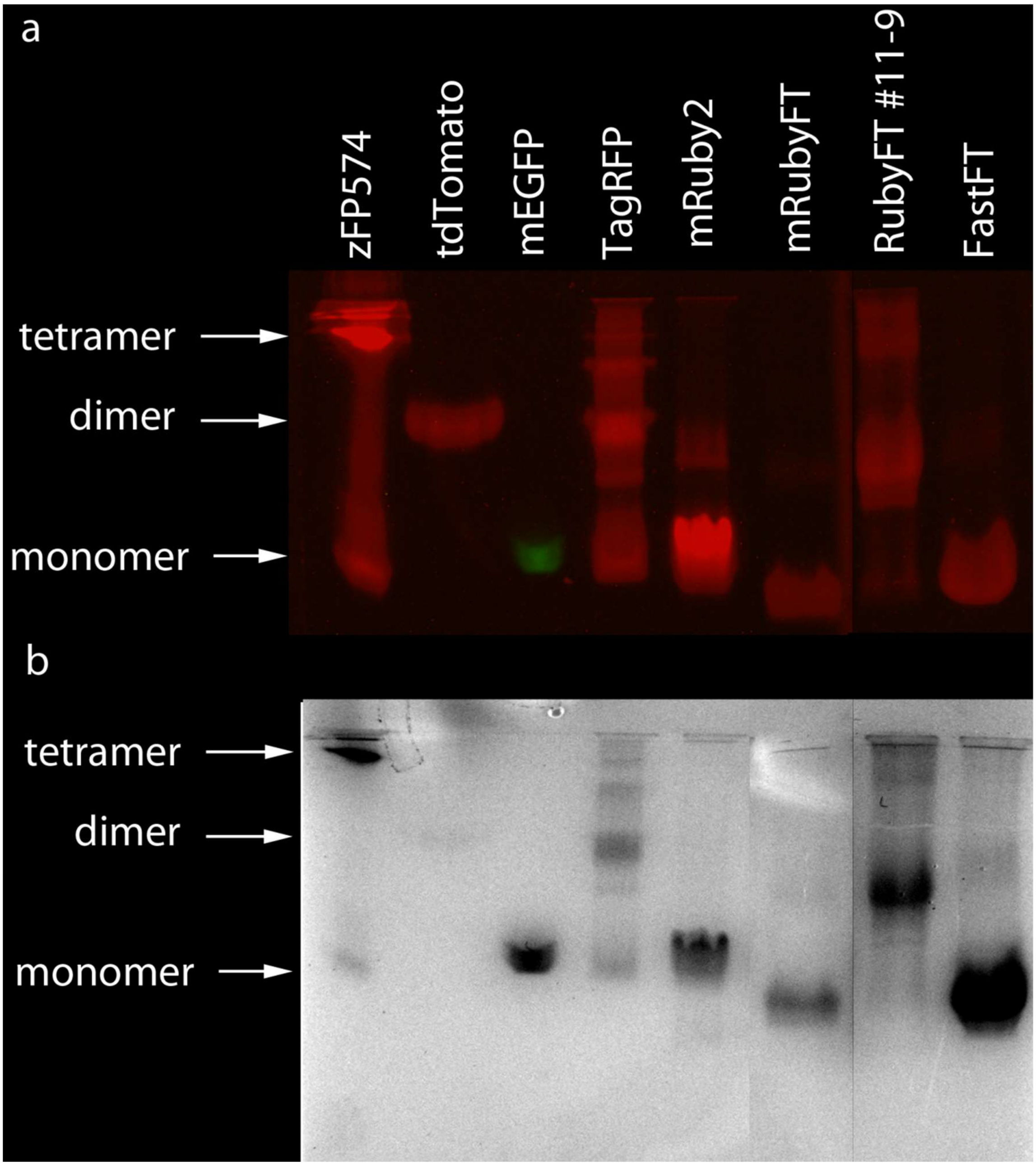

Figure S3. Semi-native polyacrylamide gel with dimeric RubyFT#11-9 and monomerized mRubyFT timers and control zFP754 , tdTomato, mEGFP, TagRFP, mRuby2 and FastFT proteins. 100-235 μg of freshly purified fluorescent proteins were loaded in 20 μl aliquots onto a semi-native 12.5% polyacrylamide gel containing 0.5% sodium dodecyl sulfate (SDS). The gel was run at 100 Volts, 25 °C. mEGFP, tdTomato and zFP574 were loaded as monomer, dimer and tetramer protein standards, respectively. (a) In the fluorescent channels, the gel was photographed using a Leica M205FA fluorescence stereomicroscope. (b) After fixation and staining with 1% Panseau S, the gel was photographed in visible light using the G: Box Chemi-XT4 GENESys system.

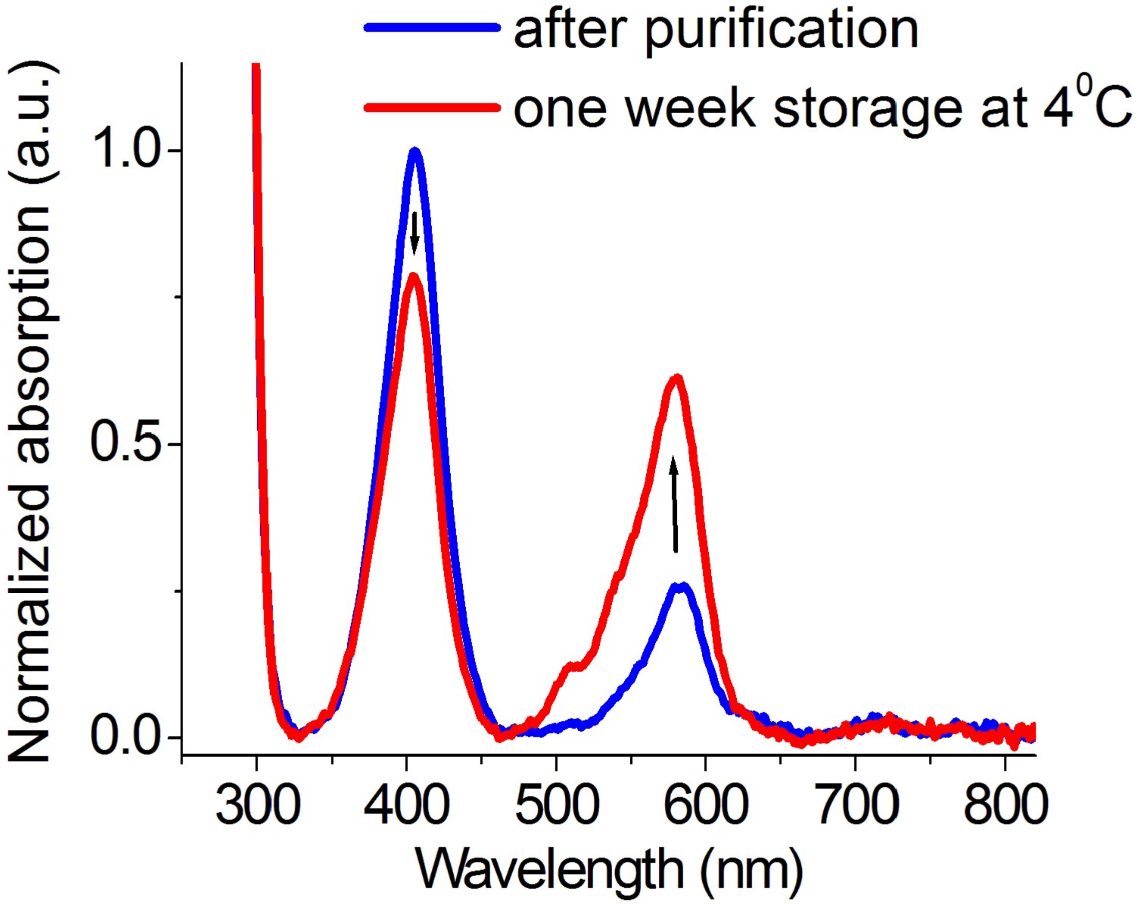

Figure S4. Changes in absorption of the purified mRubyFT protein after one week storage at 4 degrees.

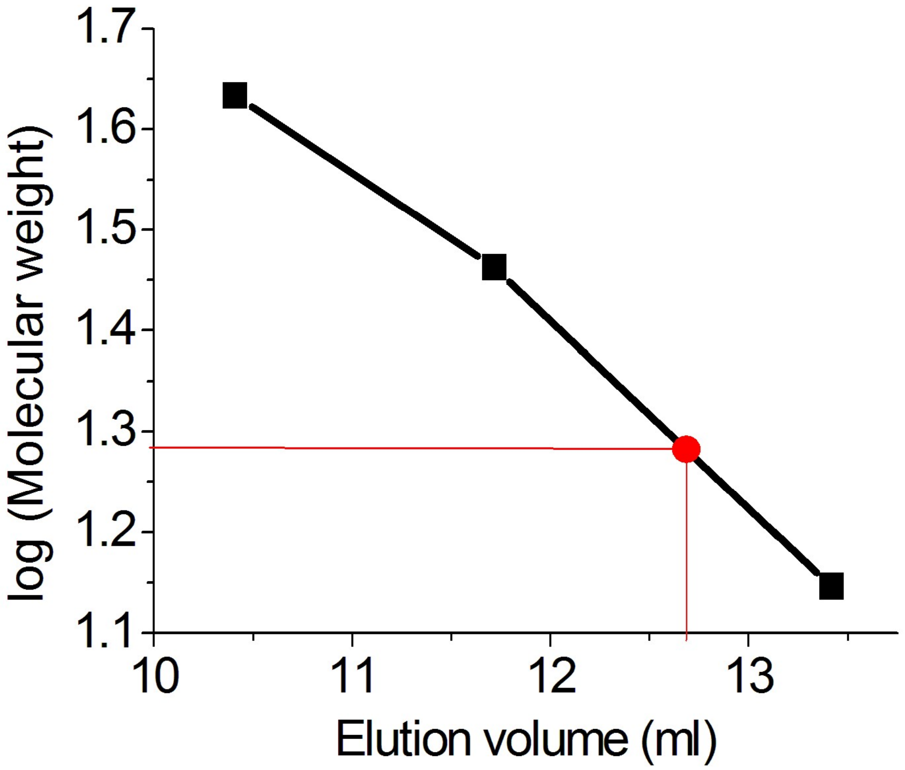

Figure S5. Fast protein liquid chromatography of mRubyFT protein. mRubyFT was eluted in 20 mM Tris-HCl (pH 7.80) and 200 mM NaCl buffer as shown in Figure 2e. The molecular weight of mRubyFT was calculated from the dependence of logarithm of control molecular weights vs. elution volume.

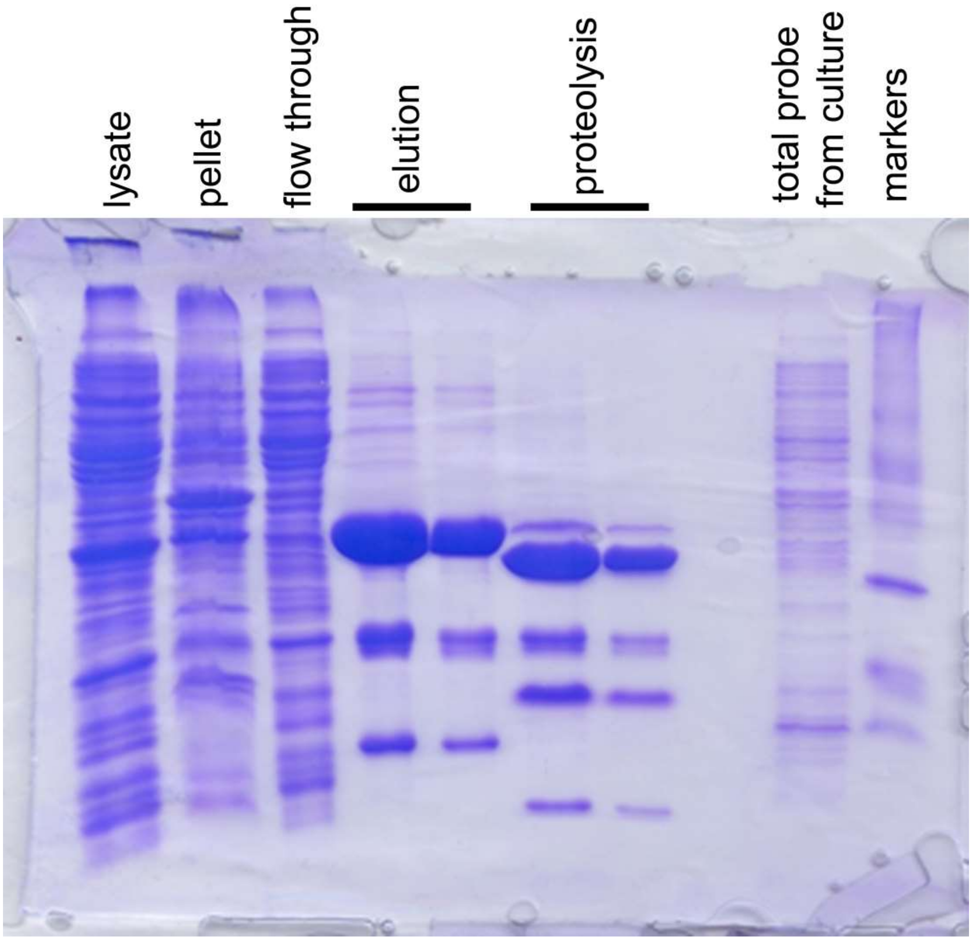

Figure S6. Results of electrophoresis in 15% PAGE, 1% SDS of the samples taken at different stages of the the mRubyFT timer purification.

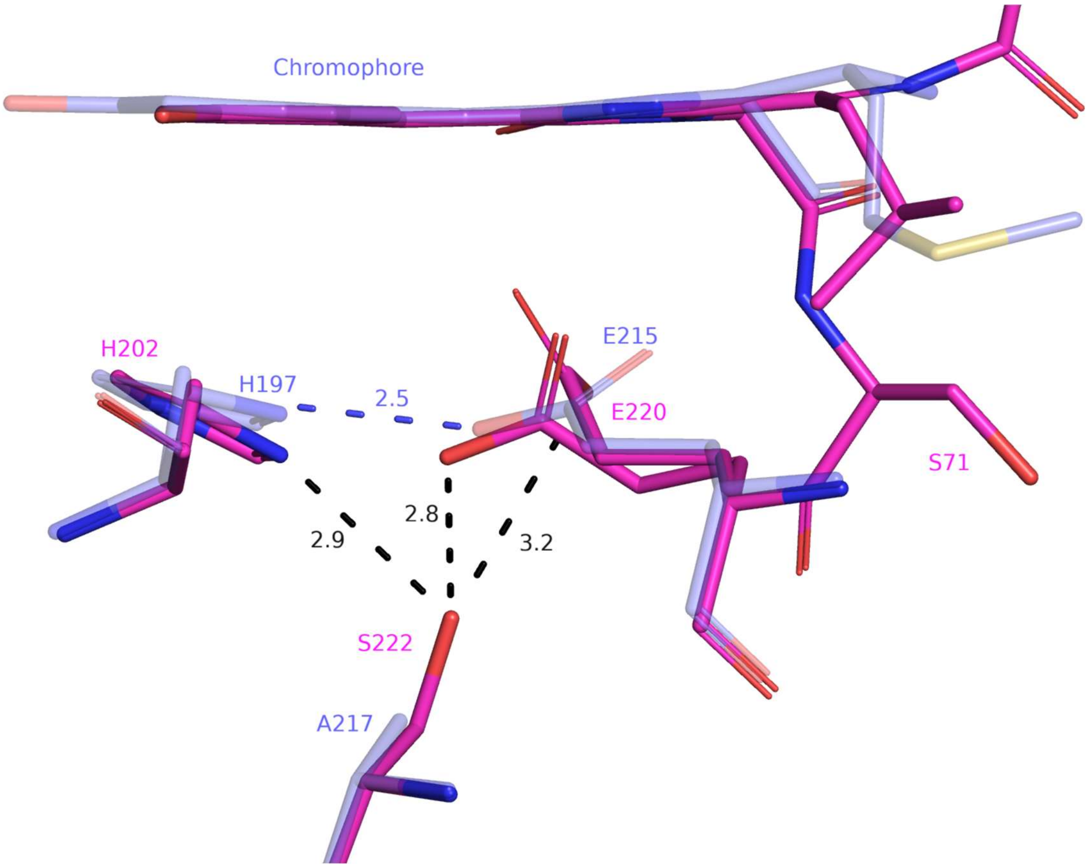

Figure S7. Effect of A222S substitution on H202 side chain orientation in mRubyFT. The structures of mRubyFT (pink) and mRuby (blue) are superimposed by their Cα atoms. Hydrogen bonds are shown as dashed lines (colored as black from mRubyFT and blue – for mRuby) with the correspondence distances labeled.
